# *GmAGL1*, a MADS-Box Gene from Soybean, Is Involved in Floral Organ Identity and Fruit Dehiscence

**DOI:** 10.3389/fpls.2017.00175

**Published:** 2017-02-09

**Authors:** Yingjun Chi, Tingting Wang, Guangli Xu, Hui Yang, Xuanrui Zeng, Yixin Shen, Deyue Yu, Fang Huang

**Affiliations:** ^1^National Center for Soybean Improvement, Key Laboratory of Biology and Genetic Improvement of Soybean Ministry of Agriculture P.R. China, State Key Laboratory of Crop Genetics and Germplasm Enhancement, Nanjing Agricultural UniversityNanjing, China; ^2^College of Agro-grassland Science, Nanjing Agricultural UniversityNanjing, China

**Keywords:** *AGAMOUS-LIKE1*, soybean, fruit dehiscence, floral organ identity genes, protein interactions

## Abstract

MADS-domain proteins are important transcription factors involved in many aspects of plant reproductive development. In this study, a MADS-box gene, *Glycine max AGAMOUS-LIKE1* (*GmAGL1*), was isolated from soybean flower. The transcript of *GmAGL1* was expressed in flowers and pods of different stages in soybean and was highly expressed in carpels. GmAGL1 is a nucleus-localized transcription factor and can interact directly with SEP-like proteins in soybean flowers. Ectopic overexpression of *GmAGL1* resulted in the absence of petals in *Arabidopsis*. Moreover, morphological changes in the valves were observed in 35S:GmAGL1 *Arabidopsis* fruits that dehisced before the seeds reached full maturity. GmAGL1 was found to be sufficient to activate the expression of *Arabidopsis ALC, IND, STK, SEP1*, and *SEP3*. Therefore, our data suggest that *GmAGL1* may play important roles in both floral organ identity and fruit dehiscence.

## Introduction

Angiosperms, or flowering plants, are the most diverse and numerous groups of plants. Despite their diversity, the most remarkable features distinguishing them from gymnosperms are their flowers and the production of fruits that contain seeds. Most flowers are composed of four types of floral organs with external sterile organs (sepals and petals) surrounding the reproductive structures (stamens and carpels) located in the center (Robles and Pelaz, 2005). The fertilized carpels give rise to the fruits, which protect the developing seeds and ultimately disperse the mature seeds into the environment. The vast majority of crops in the world are angiosperms, therefore, the regulation of flower and fruit development directly affects the economic benefits in agricultural production. Understanding the genetic factors regulating flower and fruit patterning may help to improve crop breeding.

Flower development has been the subject of intensive study for over the past 20 years. These studies led to the establishment of the well-known ABCDE model that explained the genetic regulation of floral organ identity determination. This model proposes that five classes of genes (A, B, C, D, and E) act in a combinatorial way to specify the distinct floral organs (Theissen and Saedler, 2001). The A+E protein complex determines sepals, the A+B+E complex specifies petals, the B+C+E complex specifies stamens, the C+E complex specifies carpels and the D+E complex determines the ovules (Colombo et al., 1995; Theissen and Saedler, 2001). In *Arabidopsis*, the class A genes are *APETALA1/2* (*AP1/2*) (Mandel et al., 1992; Jofuku et al., 1994); the class B genes are *AP3* (Jack et al., 1992) and *PISTILLATA* (*PI*) (Goto and Meyerowitz, 1994); the class C gene is *AGAMOUS* (*AG*) (Yanofsky et al., 1990); the class D genes are *SEEDSTICK* (*STK*) and *SHATTERPROOF1/2* (*SHP1/2*) (Favaro et al., 2003); and the class E genes are *SEPALLATA1/2/3/4* (*SEP1/2/3/4*) (Pelaz et al., 2000). Interestingly, all of these floral organ identity genes except *AP2* belong to the MADS-box gene family. Moreover, orthologs of these genes have been found in many other species, such as other eudicots, monocots, and even in gymnosperms (Robles and Pelaz, 2005; Arora et al., 2007; Katahata et al., 2014).

The genetic networks controlling fruit patterning are well-characterized in many plants (White, 2002; Xue et al., 2012; Karlova et al., 2014). In the model plant *Arabidopsis*, the fruit is a dry pod derived from two fused carpels called a silique. During dry fruit development, one main process is the differentiation of tissues required for dehiscence (Robles and Pelaz, 2005). Many genes related to fruit dehiscence have been identified. The MADS-box transcription factors SHP1 and SHP2 (previously named AGL1 and AGL5) are the primary regulators of dry fruit dehiscence (Liljegren et al., 2000). Although SHP genes have been shown to specify carpel identity in a transcriptional complex (Favaro et al., 2003), they are best known for their functions in the differentiation of the dehiscence zone and the lignification of adjacent cells (Liljegren et al., 2000). Mutations in the SHP genes lead to indehiscent fruits, thus, inhibiting the seed dispersal process. SHP genes act at the top of the genetic regulatory hierarchy in valve margin formation and positively regulate INDEHISCENT (IND) and ALCATRAZ (ALC), which are two bHLH transcription factors also required for correct valve margin development (Rajani and Sundaresan, 2001; Liljegren et al., 2004). The expression of *SHP1, SHP2, IND*, and *ALC* in the valve margins is negatively controlled by FRUITFUL (FUL). As a MADS-box transcription factor, FUL is responsible for both ovary growth and valve cell differentiation (Ferrandiz et al., 2000; Liljegren et al., 2004).

Soybean (*Glycine max* [L.] Merr.) is an economically important global crop and it is now the main source of vegetable oil and protein. The development of flowers and pods directly affects seed yield and quality. Additionally, soybean is a self-pollinated crop, the anther and stigma of which are enclosed by the wing flap and keel. This floral morphology causes difficulties in cross-pollination and prevents crossing within or between individuals, which is unfavorable for soybean hybrid breeding (Huang et al., 2014). Therefore, genetic modification of the perianth is a priority in soybean hybrid breeding. The studies on MADS-box genes related to floral morphology will facilitate the production of valuable plant materials that have potential applications in soybean hybrid breeding. Based on a previous study on gene expression analysis by Affymetrix Gene Chip (Huang et al., 2009), a *Glycine max AGAMOUS-LIKE1* gene (Probe ID: Gma.11881.1.A1_at) was found to predominantly accumulate in soybean flowers and pods, indicating its role in floral organs and fruit development (Ma et al., 1991; Flanagan et al., 1996). We isolated *GmAGL1* from soybean flower and characterized its spatial and temporal expression patterns. As a MADS domain protein, GmAGL1 is a nucleus-localized transcription factor and functions in a multimeric complex with SEP-like proteins. GmAGL1 was sufficient to activate the expression of *ALC, IND, STK, SEP1*, and *SEP3* in *Arabidopsis*. The ectopic overexpression of *GmAGL1* resulted in an abnormal floral organ identity in *Arabidopsis*, in which the petals were completely absent. Moreover, morphological changes in the valves were observed in 35S:GmAGL1 fruits that dehisced before the seeds reached full maturity.

## Materials and Methods

### Plant Materials and Growth

Soybean plants (cv. Jackson) were grown under field conditions in Nanjing Agricultural University, China. Young leaves, roots, stems and shoot apical meristems (SAMs) were collected at the third euphylis expanding stage. Flowers were harvested at different stages from tiny buds to mature flowers. Four types of floral organs were obtained from mature flowers. The developing pods were harvested at 5, 10, 20, 30, and 40 DAF (day after flowering). Seeds at 20 DAF were dissected to collect the seed coat, embryo and cotyledon under a dissection microscope with surgical blades and tweezers. All of these samples were lyophilized and stored at -80°C until used.

*Arabidopsis thaliana*, ecotype Columbia-0, was used for *GmAGL1* ectopic expression experiments. All plants were grown in a growth room at 22°C with 16 h light/8 h dark.

### Isolation of GmAGL1 Gene by RACE

Based on the sequence information from NCBI^1^, the fragment (GenBank accession No: AW433203) was found to have an incomplete ORF. The rapid amplification of cDNA using the end (RACE) technique was employed to identify both the 3′- and 5′-ends of *GmAGL1* cDNA with a SMART^TM^ RACE cDNA Amplification Kit (Clontech, USA). The 3′-end was amplified from flower cDNA by two nested PCR reactions, using the gene specific primer 3′-GSP (5′-GAGAAAGCACAACAACGGCAACAG-3′) for the first round PCR and another gene specific primer 3′-NGSP (5′-GCGAGTCAACCATACCTCCA-3′) for nested PCR. For 5′-end amplification, the two primers used were 5′-GSP (5′-TGGAGGTATGGTTGACTCGCACA-3′) and 5′-NGSP (5′-CCGTTGTTGTGCTTTCTCGTGTTC-3′). All the PCR products were gel purified, cloned into pGEM^®^-T easy vectors (Promega, USA) and sequenced (Invitrogen, Shanghai). According to the RACE results, the primer pair (sense: 5′-GCATAACACCAAAGAACTAC-3′ and anti-sense: 5′-TCACGAAACATAGGACGATT-3′) was used to isolate the intact ORF of GmAGL1 by RT-PCR.

### Sequence and Phylogenetic Analysis

The ORF of *GmAGL1* and its deduced protein sequence were analyzed by BioXM (ver 2.6). Conserved domains were analyzed by SMART^2^. The sequences of another published MADS-box genes were obtained from NCBI using BLASTP^3^. Multiple alignments were conducted with ClustalX 2.0 and viewed with GeneDOC. Sequences selected for the phylogenetic analysis were GmAGL1, the ABCDE classes of MADS-box proteins from *Arabidopsis* and previously reported AG homologs from several species. The accession numbers of the protein sequences are listed in Supplementary S2. The ML (Maximum Likelihood) phylogenetic tree was constructed using MEGA 6.0 with the following parameters: bootstrap method with 1,000 replications, “Jones-Taylor-Thornton (JTT)” as the substitution model, “complete deletion” for gaps/missing data treatment and other parameters with a default value.

### Gene Expression Analysis

Total RNA was isolated using an RNA Plant Extraction Kit (TIANGEN, China) and reverse transcribed by AMV reverse transcriptase (Takara, Japan) as described in the manufacturer’s instructions. *GmAGL1* specific primers were synthesized as follows: sense: 5′-GCTGAACACGAGAAAGCACA-3′; anti-sense: 5′-GGCACTCTCCTTCACGAAAC-3′. Semi-quantitative RT-PCR assay was performed as previously described (Huang et al., 2006). Real-time PCR was carried out with a Bio-RAD iQ5 real-time PCR system (Bio-Rad, USA) using SYBR^®^ Green Real-time PCR Master Mix QPK-201 (Toyobo Japan). The Tubulin gene (GenBank accession No: AY907703) was quantified to normalize the amount of total transcript. The relative expression of *GmAGL1* was calculated according to the method of 2^-ΔΔCt^ (Livak and Schmittgen, 2001).

For the expression analysis of MADS-box genes in transgenic *Arabidopsis*, total RNA was prepared from the flowers of the wild-type and 35S:GmAGL1 plants. The *Arabidopsis* TIP4 gene was used as an internal control. The primers for each MADS-box gene are shown in Supplementary Table S2.

### Subcellular Localization of GmAGL1

The coding region of *GmAGL1* was amplified with a sense primer (5′-CTAGTCTAGAATGGAATTTCCCAACGAAGC-3′) containing an Xba I site and an anti-sense primer (5′-CGCGGATCCGACAAGTTGAAGAGCAGTCTGGTC-3′) containing a BamH I site. The PCR product was correctly inserted into the Xba I and BamH I sites of vector pAN580, generating a GmAGL1:GFP in-frame fusion protein. This construct was then introduced into *Arabidopsis* leaf protoplasts via PEG-mediated transformation. The protoplasts were incubated under weak light for 12 h to 16 h and observed with a LSM 700 exciter confocal laser scanning microscope (Carl Zeiss). The excitation wavelength used for GFP was 488 nm.

### Ectopic Expression in *Arabidopsis*

The plant overexpression vector pMDC32 was used for *Arabidopsis* transformation and contained a double 35S CaMV promoter. The *GmAGL1* ORF from the start to stop codons was cloned into pMDC32 by Gateway^TM^ Technology (Invitrogen Shanghai). Col-0 plants were transformed with this construct by *Agrobacterium tumefaciens* using the floral dip method. Transgenic seeds were germinated on solid MS medium containing 20 μg/mL Hygromycin B. Resistant seedlings were transferred to soil and further analyzed by PCR and qRT-PCR.

### Scanning Electron Microscopy

Fruits from wild-type and 35S:GmAGL1 *Arabidopsis* plants were vacuum infiltrated with 4% glutaraldehyde in 25 mM phosphate buffer (pH 7.0) for 10 min and fixed with fresh solution for 16 h at 4°C, washed subsequently in 25 mM phosphate buffer (pH 7.0) and incubated for 4 h in 1% osmic acid. Samples were dehydrated gradually in an ethanol series of 30, 50, 70, 80, 95, and 100% and then critical point dried in liquid CO_2_. Dried samples were covered with gold in a Nanotech sputter coater and examined with a scanning electron microscope (Philips SEM-505).

### Yeast Two-Hybrid Assay

To screen the GmAGL1 interaction proteins *in vivo*, the yeast two-hybrid (Y2H) screen of the soybean cDNA library was performed by the ProQuest^TM^ Two-Hybrid System (Invitrogen). The coding region of *GmAGL1* was recombined in a pDEST32 vector that carried a GAL4 DNA-binding domain to generate the bait construct. The Y2H cDNA library prepared from soybean flowers was used as prey and co-transformed with the bait into MaV203 yeast competent cells according to the manufacturer’s protocol. Transformants were then cultured on SC-Leu-Trp-His-master plates supplemented with 40 mM of 3-aminotriazole (3-AT). The positive clones were verified by retransformation.

### Bimolecular Fluorescence Complementation (BiFC)

The coding sequences for GmAGL1, GmSEP1 (GenBank accession No: DQ159905) (Huang et al., 2009) and GmSEP3 (GenBank accession No: AJ878424) (Huang et al., 2014), were cloned into vector pUC-SPYCE (C-YFP, 156–239 amino acid) to generate the C-terminal in-frame fusions with C-YFP, whereas GmAGL1 coding sequences were introduced into pUC-SPYNE (N-YFP, 1–155 amino acid) to form N-terminal in-frame fusions with N-YFP. The plasmids were co-transformed into onion epidermal cells to verify the interaction between proteins by gold particle bombardment, with a concentration ratio of 1: 1. Two days after bombardment, imaging of co-expressed YFP fragment signals was examined with a confocal fluorescence microscope. The primers used for bimolecular fluorescence complementation (BiFC) are shown in Supplementary Table S3.

## Results

### Isolation and Sequence Analysis of *AGAMOUS-LIKE1* from Soybean

**FIGURE 1:**
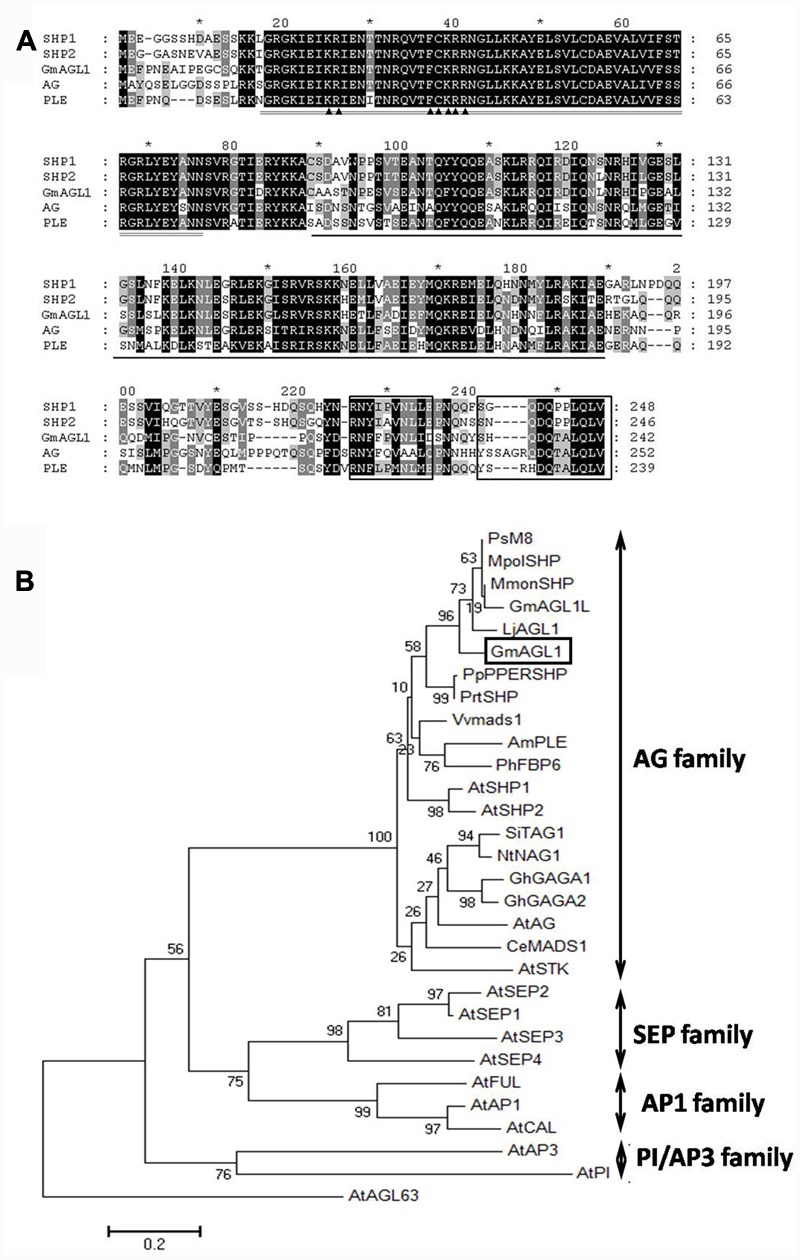
**Sequence alignment and phylogenetic analysis of GmAGL1. (A)** Clustal X alignment of the deduced amino acid sequence of GmAGL1 and other AG proteins: AG (AEE84111), SHP1 (AEE79829), SHP2 (AAU82070) and PLE (AAB25101). The highly conserved region, MADS domain, is double-underlined. The K domain is underlined. The AG motifs I and II are boxed at the C terminus. ▲ marks the bipartite NLS. **(B)** Phylogenetic relationships of ABCDE class proteins in *Arabidopsis* and AG proteins in another species. The phylogenetic tree was generated from full-length amino acid sequences by MEGA 6 using the Maximum Likelihood method. Am (*Antirrhinum majus*), At (*Arabidopsis thaliana*), Ce (*Cymbidium ensifolium*), Gh (*Gerbera hybrid*), Gm (*Glycine max*), Lj (*Lotus japonicus*), Mmon (*Medicago monspeliaca*), Mpol (*Medicago polyceratia*), Nt (*Nicotiana tabacum*), Ph (*Petunia hybrida*), Pp (*Prunus persica*), Prt (*Prunus triloba*), Ps (*Pisum sativum*), Si (*Solanum lycopersicum*), and Vv (*Vitis vinifera*). AGL63 was used as an outgroup. The accession numbers of the protein sequences are listed in Supplementary S2.

The complete *GmAGL1* including 729 bp of ORF (GenBank accession No: KY321171) was isolated from soybean flower cDNA by RT-PCR and sequenced (Supplementary S1). *GmAGL1* encodes a predicted protein of 242 amino acids with a calculated molecular mass of 27.90 kDa and a pI of 9.55. GmAGL1 contained the conserved domains that characterize MADS-box proteins: MADS domain, I domain and K domain (**Figure 1**). Although the C-terminal region was highly divergent, there were two short conserved motifs: the AG motifs I and II (**Figure 1**). Alignment analysis of amino acid sequences showed that GmAGL1 shared 86% identity with M8 (*Pisum sativum*, AAX69070) and 84% identity with LjAGL1 (*Lotus japonicus*, AAX13305). Compared with other well-known MADS-box proteins, GmAGL1 was 72% identical to SHP1/2 of *Arabidopsis* (Liljegren et al., 2000) and 78% identical to PPERSHP of Peach (Tani et al., 2007).

To determine the evolutionary relationship of GmAGL1 with other known ABCDE class proteins from *Arabidopsis*, we used the overall amino acid sequences were used for phylogenetic analysis. GmAGL1 is located in the AGAMOUS (AG) subfamily that comprises the conserved euAG and PLENA (PLE) lineages in core eudicots (**Figure 1**) (Kramer et al., 2004). GmAGL1 was highly homologous to PLE proteins in legumes and *Arabidopsis*, suggesting that it belonged to PLE lineage and may function as a class D gene in the ABCDE model.

### Expression Patterns of the *GmAGL1* during Reproductive Growth

**FIGURE 2:**
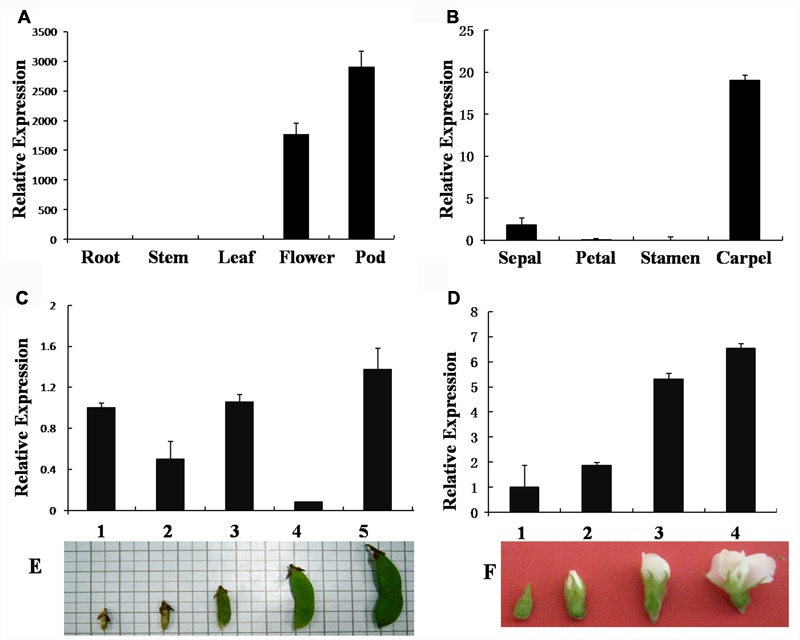
**Real-time PCR analysis of *GmAGL1* expression in different organs and at different developmental stages. (A)** Gene expression profile of *GmAGL1* in vegetative organs, flowers and pods. **(B)** Expression profile of *GmAGL1* in floral organs. **(C)** Expression profile of *GmAGL1* in developing pods at the five developmental stages (1–5) illustrated in **(E)**. **(D)** Expression profile of *GmAGL1* in developing flowers at the four developmental stages (1–4) illustrated in **(F)**. The error bars represent SD based on three replicates.

*GmAGL1* is specifically expressed in flowers and pods of different stages (**Figures 2C–F**), but not in roots, stems, and leaves (**Figure 2A**). Its expression was detected at early stages and increased gradually with the process of flower and pod development (**Figures 2C–F**). In floral organs, *GmAGL1* transcripts showed strong expression in carpels and less expression in sepals (**Figure 2B**). In the developing seed, *GmAGL1* was exclusively expressed in the seed coat, which was derived from ovule integument after fertilization (Supplementary Figure S1). The gene expression profile indicates that *GmAGL1* may play important roles in carpel and pod development.

### Nuclear Localization of GmAGL1

**FIGURE 3:**
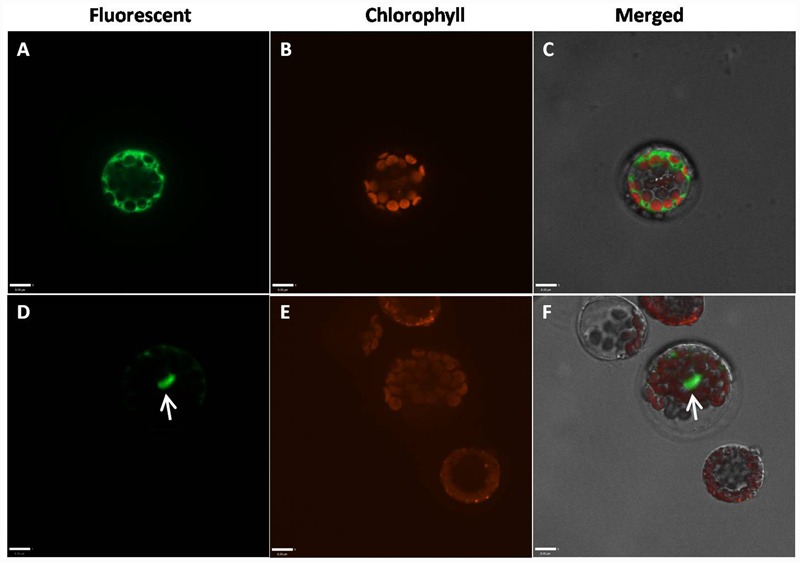
**Subcellular localization of GmAGL1.** GmAGL1-GFP fusion proteins were driven by CaMV 35S promoter and transiently expressed in *Arabidopsis* leaf protoplasts. Photographs were obtained with a confocal microscope. **(A–C)** are images of 35S:GFP as controls; **(D–F)** are images of GmAGL1-GFP. The arrow shows the location of GmAGL1. Scale bars = 8 μm.

Because the highly conserved MADS domain contains a putative nuclear localization signal (NLS) (Immink et al., 2002), GmAGL1 was predicted to be localized in the nucleus (**Figure 1**). To confirm the localization in plant cells, GmAGL1 was fused with GFP, under the control of a 35S promoter, and transiently expressed in *Arabidopsis* protoplasts. Confocal green fluorescence imaging revealed that GmAGL1–GFP fusion protein was located in the nucleus, whereas free GFPs, 35S:GFP, were distributed throughout the whole cell (**Figure 3**).

### Phenotypic Changes in *GmAGL1* Ectopic Expression Lines

**FIGURE 4:**
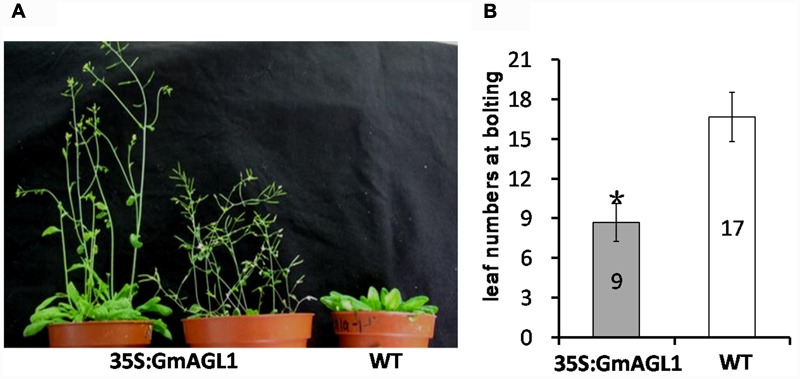
**Early flowering in 35S:GmAGL1 transgenic *Arabidopsis* plants. (A)** Image of wild-type plants (WT) and transgenic plants (35S:GmAGL1) overexpressing GmAGL1 at 6 weeks after germination. **(B)** Comparison of leaf numbers at bolting between 35S: GmAGL1 and WT plants. Values correspond to the average leaf numbers at bolting. Error bars represent the standard deviation. An asterisk indicates a significant difference (*t*-test, *P* < 0.01) between transgenic and wild-type plants.

**FIGURE 5:**
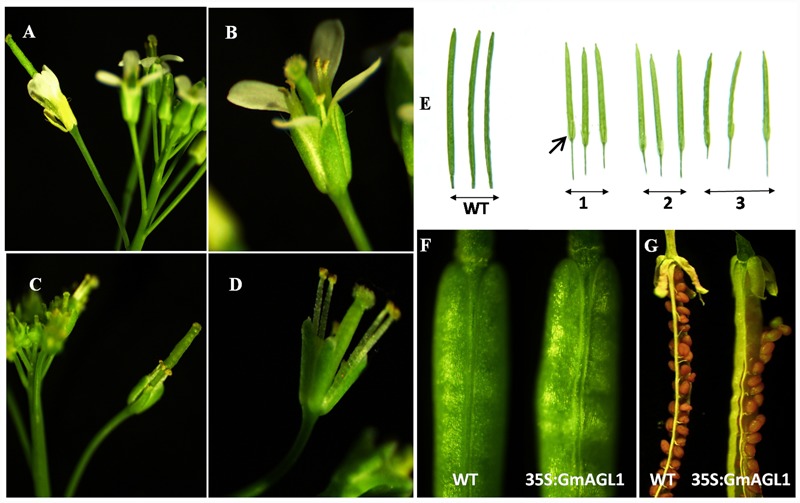
**Abnormal morphology of flowers and siliques in 35S:GmAGL1 transgenic *Arabidopsis* plants. (A,B)** The wild-type flowers at anthesis showing four petals surrounding the stamen and stigma. **(C,D)** 35S:GmAGL1 flowers. Note the complete absence of petals. **(E)** The siliques from three 35S:GmAGL1 lines (1–3) are short and yellowish-green with unabscised sepals, compared to those from wild-type (WT). **(F)** The closer view of the siliques. **(G)** The silique of 35S:GmAGL1 dehisced before the valves turned yellow and the seeds reach full maturity, which was much earlier than in wild-type (WT).

To characterize the function of GmAGL1 further, we examined transgenic *Arabidopsis* plants that were constitutively expressed *GmAGL1* under the control of the cauliflower mosaic virus 35S promoter (35S:GmAGL1). The overexpression of *GmAGL1* significantly affected the development of the transgenic *Arabidopsis*, which flowered substantially earlier than the flowers of wild-type plants (**Figure 4A**). The number of leaves produced in the transgenic lines at bolting was significantly different from that in the wild-type plants. The transgenic lines produced on average 9 leaves before flowering, whereas wild-type plants produced approximately 17 leaves (**Figure 4B**). Flowers from plants highly expressing GmAGL1 showed an abnormal morphology, in which all the petals were absent (**Figures 5A–D**). After fertilization, the senescence and abscission of sepals were significantly delayed in transgenic plants, and green unabscised sepals were observed even in the matured siliques (**Figure 5E**).

**FIGURE 6:**
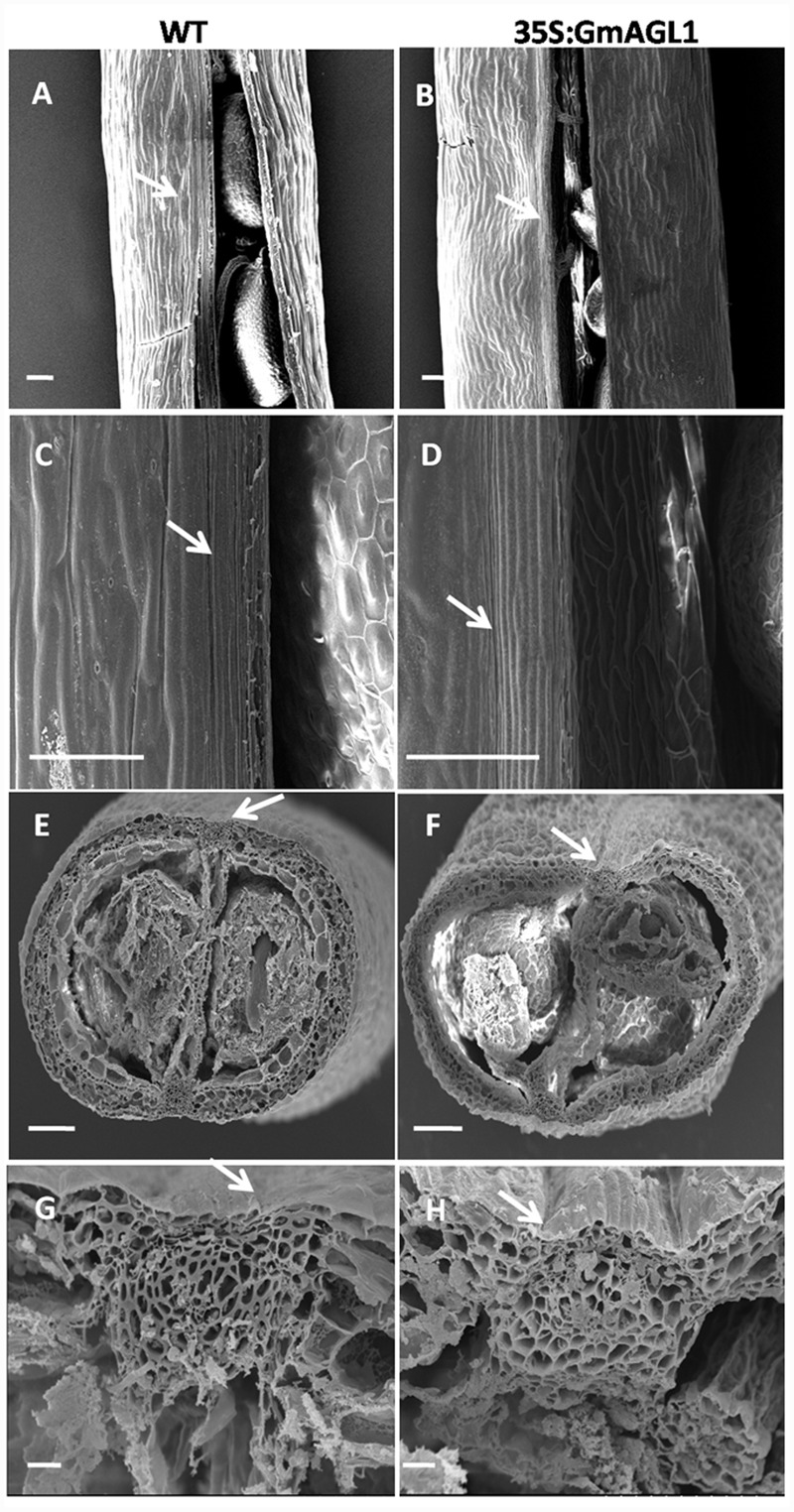
**Scanning electron micrograph of 35S:GmAGL1 transgenic *Arabidopsis* silique. (A–D)** The epidermal cells morphology in silique of wild-type and 35S:GmAGL1. **(E–H)** The cross sections cellular structures in siliques of wild-type and 35S:GmAGL1 plants. Arrows mark the dehiscence zones. Scale bar = 100 μm.

Moreover, phenotypic differences between the developing fruits of 35S:GmAGL1 and wild-type plants were also observed. 35S:GmAGL1 fruits were shorter in length and appeared yellowish-green compared to the long and dark green color of wild-type fruits (**Figure 5E**). The valve margins of 35S:GmAGL1 fruits were more remarkable than those of wild-type fruits (**Figure 5F**). To gain a clear observation, scanning electron microscopy was used to examine the morphological changes in the fruits. The outer epidermis of 35S:GmAGL1 fruit valves differed from that of wild-type fruit valves. The valve margins were significantly wider and thinner compared to wild-type fruits (**Figures 6A–D**). The microscopic cross section of the transgenic siliques cracking are showed a less condensed structure and reduced thickness (**Figures 6E–H**). In the premature fruits, dehiscence occurred in the valve regions while the seeds were still fresh and green, resulting in early seed dispersal (**Figure 5G**). These gain-of-function analyses suggest that GmAGL1 has a direct role in promoting valve development and regulating fruit elongation and dehiscence.

### Expression Analysis of Genes in Transgenic *Arabidopsis*

**FIGURE 7:**
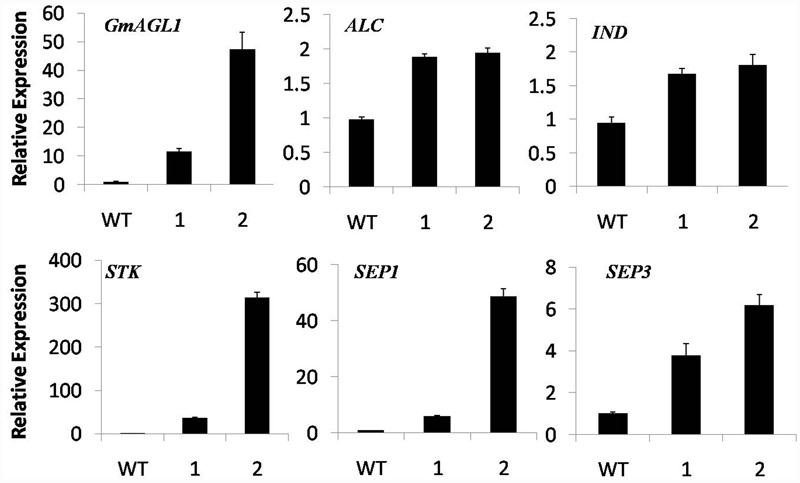
**Real-time PCR analysis of flowering and fruit dehiscence related gene expression in 35S:GmAGL1 transgenic *Arabidopsis* flowers.** The TIP4 gene was used as an internal control. The primers for quantitative RT-PCR are shown in Supplementary Table S2. The error bars represent SD based on three replicates.

GmAGL1 was localized to the nuclear compartments of plant cells, indicating that it might regulate gene expression as a transcription factor. Based on the function of GmAGL1, as revealed by its constitutive expression, we studied the expression of five genes involved in flowering and fruit development in the 35S:GmAGL1 flowers. It was observed that the *Arabidopsis* homologs of the fruit dehiscence related genes, *ALC, IND* and *STK*, showed more accumulation in 35S:GmAGL1 compared to wild-type plants (**Figure 7**). As will be described later in the Y2H screen, GmAGL1 interacted with SEP homologs. The expression levels of both *SEP1* and *SEP3* were increased in the 35S:GmAGL1 *Arabidopsis* (**Figure 7**). These results suggested that GmAGL1 might directly regulate the expression of these genes to control flower development and fruit dehiscence.

### Screening of GmAGL1 Interaction Proteins in Flowers

We applied a GAL4-based experiment to screen for the proteins that interact directly with GmAGL1. As shown in Supplementary Figure S2, the yeast cells containing BD-GmAGL1 could not grow on the SC-Leu-Try-His+40 mM 3AT medium, nor could the negative self-activation control. This suggests that GmAGL1 exhibits no transactivation activity and can be used as bait to perform a Y2H screen. As a result, we identified four proteins as interacting proteins of GmAGL1, which were named Interaction Protein 1 to Interaction Protein 4 (IP1∼4) (Supplementary Table S1). Two of these were SEP1-like proteins (IP1/3), one was a SEP3-like protein (IP2), and one was a putative CHUP1 protein (IP4). IP1 (Glyma18g50900), IP2 (Glyma08g11120), and IP3 (Glyma08g27670) were highly similar to homologs of SEP in the MADS-box gene family. This result suggests that GmAGL1 and SEP-like proteins in soybean can interact in specific manners and form macromolecular complexes to regulate flower and fruit development.

### GmAGL1 Interacts with Soybean SEP Homologs in the Nucleus

**FIGURE 8:**
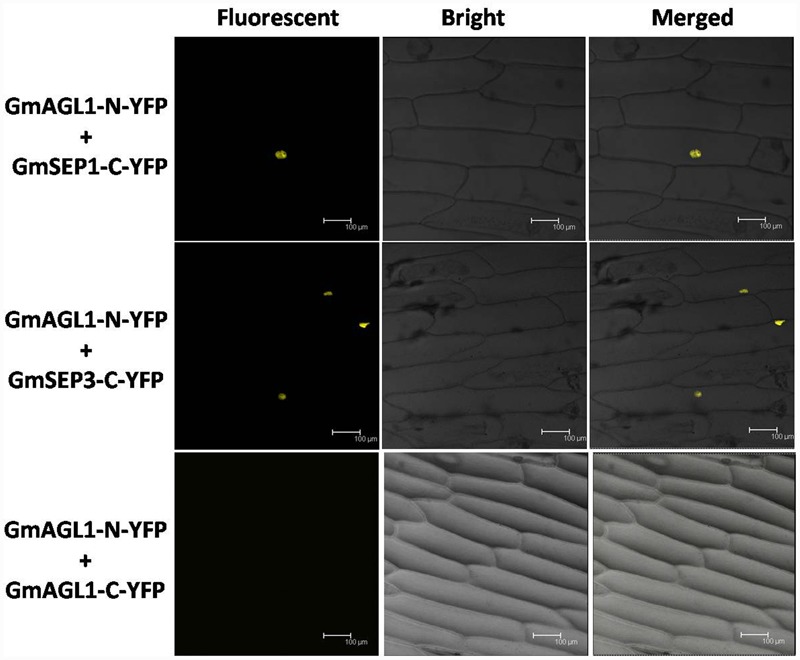
**Bimolecular fluorescence complementation (BiFC) analysis of GmAGL1 interactions *in planta*.** Fluorescence was observed in the nuclear compartment of onion epidermal cells and results from complementation of the N-terminal part of the YFP fused with GmAGL1 (GmAGL1-N-YFP) with the C-terminal part of the YFP fused with soybean SEP homologs (GmSEP1/3-C-YFP). No fluorescence was observed when GmAGL1-N-YFP was co-expressed with GmAGL1-C-YFP. Bright-field images, YFP epifluorescence images and overlay images of the same cells are shown. Scale bar = 100 μm.

The interactions between GmAGL1 and SEP homologs were further confirmed *in vivo* by BiFC assay. Two SEP functional homologs studied in soybean (Huang et al., 2009, 2014) were inserted into pUC-SPYCE (GmSEP1/3-C-YFP). Using particle bombardment, GmAGL1-N-YFP and GmSEP1/3-C-YFP were co-transformed into onion epidermal cells. Fluorescence confocal microscopy showed that BiFC signals were present in the nuclear compartments of transformed cells (**Figure 8**). GmAGL1-N-YFP and pUC-SPYCE, pUC-SPYNE and GmSEP1/3-C-YFP, pUC-SPYNE and pUC-SPYCE – as negative controls – were separately bombarded into onion epidermal cells, after which no fluorescence was detected (Supplementary Figure S3). These experiments demonstrated that GmAGL1 interacted with soybean SEP homologs in the nucleus.

### GmAGL1 Cannot Form Homodimers

The BIFC system was also used to detect whether GmAGL1 can form homodimers. Full-length GmAGL1 sequences were inserted into pUC-SPYNE and pUC-SPYCE to construct the recombinant plasmids GmAGL1-N-YFP and GmAGL1-C-YFP, respectively, where GmAGL1 was in-frame fused with the N-terminus and C-terminus of YFP. GmAGL1-N-YFP and GmAGL1-C-YFP were co-expressed in onion epidermal cells through particle bombardment, but no YFP signals were found (**Figure 8**). These results indicated that GmAGL1 did not interact with itself in plant cells, i.e., GmAGL1 cannot form homodimers.

## Discussion

The MADS-box transcription factor family is a large family that controls flower and fruit patterning in plants. To date, many members of this family have been identified and extensively studied in different plant species. However, much less is known about their functions in the developmental processes of soybean (Huang et al., 2014). In a previous study, genome wide expression profiles of soybean genes were investigated by Affymetrix Gene Chip in roots, leaves, flowers and pods (Huang et al., 2009). Some MADS-box genes were found to be primarily expressed in both flowers and pods. The roles of these genes are still not clear and require further functional analysis. In this study, we functionally characterized a soybean MADS-box gene, *GmAGL1*.

GmAGL1 is a MIKC^c^ type MADS-box protein encoded by genomic DNA, as it possesses a modular structure where the MADS (M) domain is followed by an intervening (I), a keratin (K) and a C-terminal (C) domain (Theissen et al., 1996; Kaufmann et al., 2005). Two short regions of high conservation, the AG motifs I and II, were identified in the variable C domain. These two motifs are conserved in the AG subfamily (Kramer et al., 2004). Ectopic expression of an AG protein lacking the C-terminal produced *ag*-like flowers in transgenic *Arabidopsis*, indicating that these AG motifs are required for the correct function of AG proteins in plant development (Mizukami et al., 1996). Phylogenetic analyses also showed that GmAGL1 was clustered into the AG subfamily and closely allied with the PLE lineage. Members of the AG subfamily have been characterized from angiosperms as master regulators of stamen, carpel and ovule identities. They play important roles after fertilization in the developing fruits and seeds. These studies also demonstrated that *AG*-like genes retain functional conservation within flowering plants (Dreni and Kater, 2014). The functional conservation of *AG* homologs suggests that *GmAGL1* might have central roles in regulating soybean floral organ identity and pod development.

Previous results showed that *GmAGL1* was exclusively expressed in flowers and pods. We found that *GmAGL1* was specifically expressed in carpels, pod walls and seed coats, but only weak expression was seen in sepals. The transcript was detected at all stages of flower and pod development. The expression pattern of *GmAGL1* was similar to that observed for other *AG*-like genes. For instance, the transcript of *PPERSHP* was detected primarily in carpels and accumulated throughout fruit development from full anthesis until fruit harvest (Tani et al., 2007). *AGL1* was preferentially expressed in particular regions of the gynoecium and ovule, only during and after floral development (Flanagan et al., 1996). The spatial and temporal expression profiles of *AG*-like genes are closely aligned with their conserved functions in carpel and pod development.

Consistent with a role as a transcription factor, GmAGL1 was localized in the nucleus and contained putative DNA-binding domain in the N-terminus. Nevertheless, none of the putative motifs related to transcriptional activation were found in the conserved domain analysis of GmAGL1. The full length of GmAGL1 also exhibited inactivity in the self-activation test in the Y2H system. Thus far, the ability of transactivation has not been described in AGL1 proteins. Interestingly, the proteins that directly interact with GmAGL1 were obtained from a soybean flower cDNA library by a yeast two-hybrid screen. It was also reported that AP1 and SEP3 added transcriptional activator activity to PI–AP3 and AG by the formation of complexes (Honma and Goto, 2001). Therefore, we predict that GmAGL1 might allow transcriptional regulation of target genes as an activator in a combinatorial way by interacting with other factors.

It is well-known that MADS domain proteins do not exert their functions as monomers, but rather they form multimeric protein complexes with other proteins (de Folter et al., 2005; Immink et al., 2010). In this study, a screen of a cDNA expression library with the yeast two-hybrid GAL4 system was performed to unravel the protein–protein interaction network for GmAGL1. As a result, three putative MADS-box transcription factors were identified as GmAGL1 interaction partners from soybean flowers. Interestingly, all these proteins belonged to the SEP subfamily, indicating that the function of GmAGL1 in promoting carpel identity may be based on a biochemical interaction with SEP proteins. In *Arabidopsis*, three SEP factors, SEP1, SEP2 and SEP3, which are closely related and functionally redundant, were necessary to determine the identities of petals, stamens, and carpels (Pelaz et al., 2000). In addition, protein-protein interaction studies have revealed that MADS-box proteins are dependent for their function of floral organ identity on the interaction with SEP proteins (Honma and Goto, 2001). These findings contributed to the proposal of the genetic ABCDE model for flower development, which demonstrated that the members of SEP proteins (E-class) act as bridges allowing the formation of higher order complexes (Theissen and Saedler, 2001). The study also showed that the complexes composed of AGL1 and SEP homologous proteins are probably able to promote carpel identity in the absence of AG and AP2 (Favaro et al., 2003).

To investigate the biological function *in planta*, we overexpressed *GmAGL1* in transgenic *Arabidopsis*. We observed flowers with abnormal morphology wherein the petals were absent. The senescence and abscission of sepals were delayed in the developing transgenic siliques. These observations suggested that GmAGL1 may have a similar activity to the C function gene *AG*, whose overexpression caused homeotic conversion of perianth organs by suppressing A function genes (Mizukami and Ma, 1995). In *Arabidopsis, SHP1/2* have retained the ability to substitute for *AG* activity, as the flowers constitutively expressing *SHP1* or *SHP2* showed a phenotype similar to those constitutively expressing *AG* (Liljegren et al., 2000). The introduction of 35S:SHP2 into *ag* mutants was sufficient to rescue stamen and carpel development (Pinyopich et al., 2003). Many studies have shown that the overexpression of *AG*-like genes would alter floral organ identities in the two perianth whorls (Liljegren et al., 2000; Boss et al., 2001; Vrebalov et al., 2009; Gimenez et al., 2010). In addition, GmAGL1 interacted with soybean SEP-like proteins and promoted *SEP* expression in *Arabidopsis*. SEP-like proteins are necessary to determine the identities of petals, stamens and carpels (Pelaz et al., 2000; Huang et al., 2014). GmAGL1 might interfere with the activity of petal specification genes through competition for interacting partners.

In general, fruits are derived directly from the carpels. Therefore, any mutation that affects carpel development has an effect on fruit development. *GmAGL1* is also important for fruit development. The overexpression of *GmAGL1* in *Arabidopsis*, which has a dry dehiscent fruit similar to the soybean pod, resulted in striking phenotypic effects in the 35S:GmAGL1 lines fruits, which were short, yellowish-green and early dehiscent. Microscopy revealed that valve margins were more visibly constricted in the developing transgenic fruits, which led to the dehiscence of valves before the seeds reach full maturity. In *Arabidopsis*, there has been significant progress in the molecular mechanisms of fruit dehiscence. *ALC* and *IND* encode two bHLH transcription factors related to *Arabidopsis* pod shatter, which is regulated by SHP in the genetic regulatory hierarchy (Rajani and Sundaresan, 2001; Liljegren et al., 2004). *STK* is a D-type MADS-box gene that is required for normal development of the funiculus, an umbilical-cord-like structure that connects the developing seed to the fruit, and for dispersal of the seeds when the fruit matures (Pinyopich et al., 2003). As expected, the overexpression of *GmAGL1* activated the expression of native *ALC, IND* and *STK*, which might be the molecular mechanism for the phenotypic effects of transgenic *Arabidopsis*. The functional studies of other *AG*-like genes gave very similar results, not only in *Arabidopsis* (Liljegren et al., 2000) but also in tobacco (Fourquin and Ferrandiz, 2012) and tomato (Gimenez et al., 2010), suggesting that *AG*-like genes may play a prominent role late in fruit development and dehiscence that is generally conserved in other eudicots. Pod dehiscence is a major cause of yield loss in mechanical harvesting of soybeans, especially in several countries in tropical and sub-tropical regions (Tiwari and Bhatia, 1995). Our results indicate that further analysis of the molecular network underlying fruit dehiscence may contribute to the potential genetic manipulation of pod shattering in soybean plants.

## Author Contributions

FH and DY conceived this project. YC and TW designed and performed the sequence characterization, expression profile and protein interaction of GmAGL1. GX, HY, and XZ conducted the plant transformation and microscopy analysis. YC and FH wrote the article. YS and DY contributed to scientific discussions and critical revision of manuscript. All authors reviewed the final manuscript.

## Conflict of Interest Statement

The authors declare that the research was conducted in the absence of any commercial or financial relationships that could be construed as a potential conflict of interest.

## Footnotes

1 http://www.ncbi.nlm.nih.gov

2 http://smart.embl-heidelberg.de/

3 http://www.ncbi.nlm.nih.gov/blast

## Abbreviations

BD: binding domain
GFP: green fluorescent protein
ORF: open reading frame
Y2H: yeast two hybrid
