# Supplementary material for: GmAGL1, a MADS-Box Gene from Soybean, Is Involved in Floral Organ Identity and Fruit Dehiscence
Source: Front Plant Sci. 2017 Feb 9;8:175. doi: 10.3389/fpls.2017.00175 (PMC5299006; doi:10.3389/fpls.2017.00175)
Supplement: Supplementary file 1 [file Presentation_1.PDF]

GmAGL1, a MADS-box gene from soybean, is involved in floral organ identity and fruit dehiscence

Yingjun Chi[12], Tingting Wang[1], Guangli Xu[1], Hui Yang[1], Xuanrui Zeng[1], Yixin Shen[2], Deyue Yu[1], Fang Huang[1*]

Supplementary S1 Transcript sequence of *GmAGL1* and its deduced peptide sequence.

> GmAGL1 transcript sequence
GCATAACACCAAAGAACTACAAATAGTTCTCATTGCATAGCCATAGATGGAATTTCC
CAACGAAGCAATACCAGAAGGGTGTTCACAGAAGAAAACGGGGAGGGGGAAAATAG
AAATCAAGCGGATCGAGAACACAACGAATAGGCAAGTTACCTTCTGCAAACGCCGTA
ACGGGTTGCTCAAGAAGGCTTATGAATTGTCTGTTCTGTGTGATGCTGAGGTTGCTCT
TGTTGTCTTCTCAAGCCGTGGACGCCTCTATGAGTATGCCAACAACAGTGTTAGAGGA
ACGATCGATAGGTACAAGAAAGCATGTGCTGCCTCCACAAATCCAGAATCTGTCTCT
GAAGCTAATACACAGTTTTATCAGCAGGAAGCGTCCAAATTAAAAAGACAAATCAGA
GACATTCAGAATCTAAACAGGCACATCCCTGGTGAAGCTCTTAGCTCTCTGAGTCTGA
AGGAATTAAAGAACCTGGAGAGTAGACTGGAGAAAGGTTTAAGCAGAGTTAGATCC
AGAAAGCATGAAACTTTGTTTGCCGATATCGAGTTCATGCAAAAGCGGGAAATAGAG
CTGCAAAACCATAATAATTTTCTGAGAGCTAAGATAGCTGAACACGAGAAAGCACAA
CAACGGCAACAGGATATGATACCGGGAAATGTGTGCGAGTCAACCATACCTCCACAA
TCATATGACCGCAATTTCTTCCCTGTTAATCTCATAGATTCCAATAATCAATATTCACA
TCAAGACCAGACTGCTCTTCAACTTGTCTGATAAACGATTTGTAATTATGCCGCAGCC
TTTCAAGTGGTGTGGGATCCCTTAAATCGTCCTATGTTTCGTGA

> GmAGL1 amino acid sequence
MEFPNEAIPEGCSQKKTGRGKIEIKRIENTTNRQVTFCKRRNGLLKKAYELSVLCDAEVAL
VVFSSRGRLYEYANNSVRGTIDRYKKACAASTNPESVSEANTQFYQQEASKLKRQIRDIQ
NLNRHIPGEALSSLSLKELKNLESRLEKGLSRVRSRKHETLFADIEFMQKREIELQNHNNFL
RAKIAEHEKAQQRQQDMIPGNVCESTIPPQSYDRNFFPVNLIDSNNQYSHQDQTALQLV

Supplementary S2 The accession numbers of protein sequences used in the phylogenetic analysis.

AmPLE (AAB25101); AtAG (AEE84111); AtAGL63 (AAN52807); AtAP1(CAA78909); AtAP3 (AAD51903); AtCAL (AAA64789); AtFUL (AAA97403); AtPI (AAD51998); AtSEP1(AAU81996); AtSEP2 (AAA32734); AtSEP3(O22456); AtSEP4 (AAB38975); AtSHP1 (AEE79829); AtSHP2 (AAU82070); AtSTK (AAC49080); CeMADS1 (ADP00515); GhGAGA1 (CAA08800); GhGAGA2 (CAA08801); GmAGL1L (ACA24479); LjAGL1 (AAX13305); MmonSHP (AGK25045); MpolSHP (AGK25046); NtNAG1 (Q43585); PhFBP6 (CAA48635); PpPPERSHP (ABG75908); PrtSHP (AEI01160); PsM8 (AAX69070); SiTAG1 (AAA34197); Vvmads1 (AAK58564).

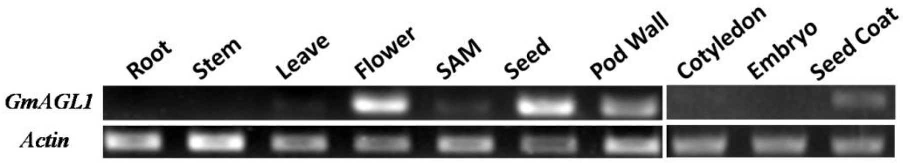

Supplementary Fig S1 Semi-quantitative RT-PCR analysis of *GmAGL1* expression in different tissues of soybean. *Actin* was used as the reference gene.

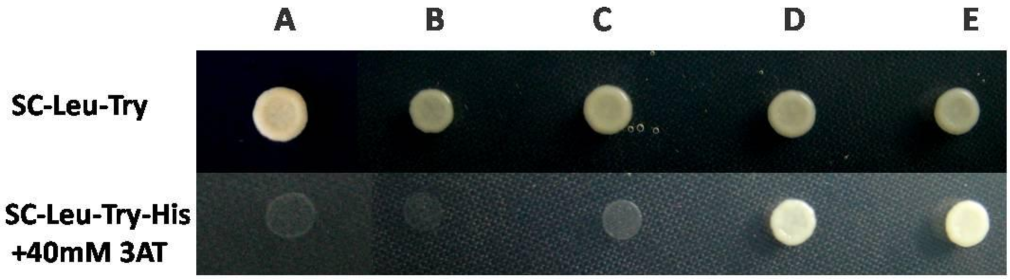

Supplementary Fig S2 Assay of GmAGL1 transcriptional activity. (A) Yeast cells were transformed by BD-GmAGL1 and pDEST22. (B) The transformations of pDEST32 and pDEST22 served as negative controls, while the transformation of pEXP™32/Krev1 with pEXP™22/RalGDS-wt or pEXP™22/RalGDS-m1 or pEXP™22/RalGDS-m2 served as strongly positive (E), weakly positive (D) and absent (C) controls, respectively. The transformants were plated on non-selective SC-Leu-Try and selective SC-Leu-Try-His + 40 mM 3AT plates for examination of growth.

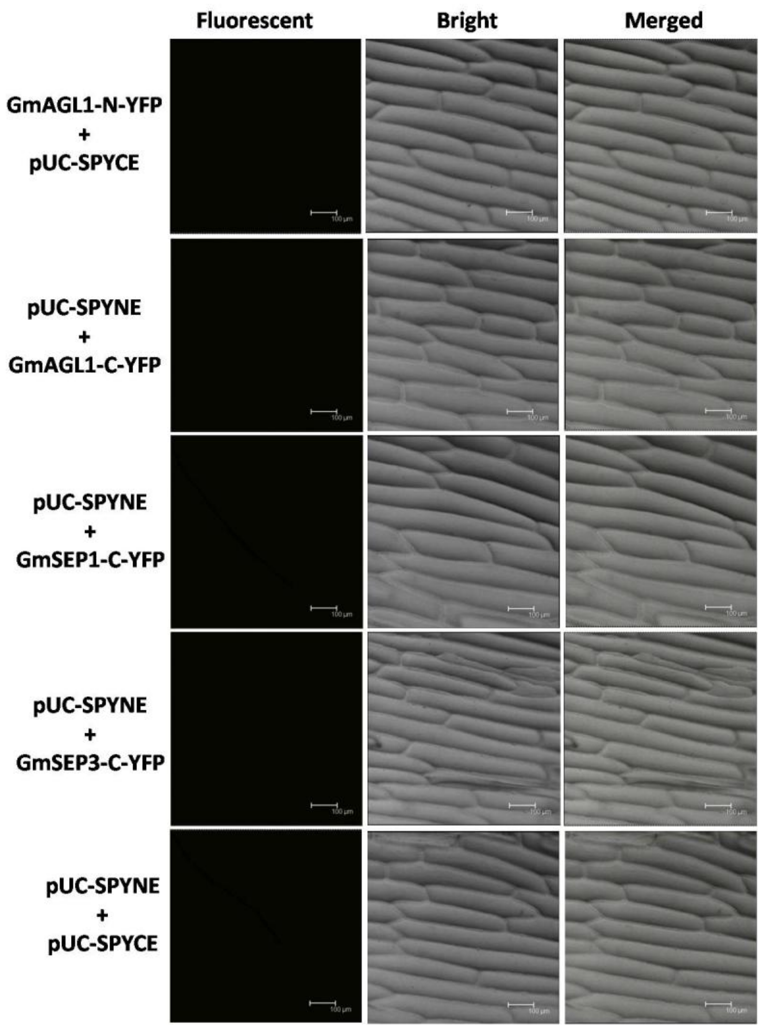

Supplementary Fig S3 Negative controls for BiFC analysis of GmAGL1 interactions *in planta*. No fluorescence was observed in the onion epidermal cells. Bright-field images, fluorescence images and overlay images of the same cells are shown. Scale bar = 100 μm.

Supplementary Table S1 GmAGL1 interaction proteins in soybean flowers.

Protein No.	Gene ID	Annotation
IP1	Glyma18g50900	MADS box protein SEP1
IP2	Glyma08g11120	MADS box protein SEP3
IP3	Glyma08g27670	MADS box protein SEP1
IP4	Glyma15g02420	Putative CHUP1 protein

Supplementary Table S2 Primers for gene expression analysis in transgenic *Arabidopsis*.

Gene Name	Accession Number	Primer Sequences
GmAGL1	AW433203	F: CCTGGAGAGTAGACTGGAGA<br>R: TGCGGTCATATGATTGTGGA
IND	NM_116229	F: AAAAGAAGCATGATGGAGCCT<br>R: GGGTTGGGAGTTGTGGTAAT
ALC	NM_126111	F: CCGACAGATTCTTTCCC<br>R: TGGTTCCAGCGAGTGAG
SEP1	NM_121585	F: AGCAAGTTCGGTCCAT<br>R: GGGTTGTCGCAGTTATT
SEP3	NM_001198152	F: TACAAAGGAGCTTGAGTCAC<br>R: CCTCTTCTTGGTTAGGGTTC'
STK	NM_179020	F: ACGCCAATAACAACATA<br>R: AAGAACCTCCATTACCAG
TIP4	NM_119592	F: AGGGTATCCAGTTGAC<br>R: ATTTGCGAAATACCG

Supplementary Table S3 Primers for BiFC constructs.

Protein Name	Primer Sequences
GmAGL1	F: GCTCTAGAATGGAATTTCCCAACGAAGC<br>R: CGCGGATCCGACAAGTTGAAGAGCAGTC
GmSEP1	F: GCTCTAGAATGGGGAGGGGAAGAGTGGA<br>R: CGCGGATCCAAGCATCCATCCAGGAATAA
GmSEP3	F: GCTCTAGAATGGGAAGGGGAAGAGTGGA<br>R: CGCGGATCCTGGTAACCATCCTGCCATGT
